# Joint use of location and acceleration data reveals influences on transitions among habitats in wintering birds

**DOI:** 10.1038/s41598-023-28937-x

**Published:** 2023-02-06

**Authors:** Jay A. VonBank, Toryn L. J. Schafer, Stephanie A. Cunningham, Mitch D. Weegman, Paul T. Link, Kevin J. Kraai, Christopher K. Wikle, Daniel P. Collins, Lei Cao, Bart M. Ballard

**Affiliations:** 1 Caesar Kleberg Wildlife Research Institute, Texas A&M University-Kingsville, Kingsville, TX 78363 USA; 2 Department of Statistics, University of Missouri, Columbia, MO 65211 USA; 3 School of Natural Resources, University of Missouri, Columbia, MO 65211 USA; 4 Department of Biology, University of Saskatchewan, Saskatoon, SK S7N 5E2 Canada; 5 Louisiana Department of Wildlife and Fisheries, Baton Rouge, LA 70808 USA; 6 Texas Parks and Wildlife Department, Canyon, TX 79015 USA; 7 U.S. Fish and Wildlife Service Region 2, Albuquerque, NM 87102 USA; 8 State Key Laboratory of Urban and Regional Ecology, Research Center for Eco-Environmental Sciences, Chinese Academy of Sciences, Beijing, 100085 China; 9 University of Chinese Academy of Sciences, Beijing, 100049 China; 10 Present Address: U.S. Geological Survey, Northern Prairie Wildlife Research Center, Jamestown, ND 58401 USA

**Keywords:** Biological techniques, Ecology, Zoology, Ecology

## Abstract

Quantifying relationships between animal behavior and habitat use is essential to understanding animal decision-making. High-resolution location and acceleration data allows unprecedented insights into animal movement and behavior. These data types allow researchers to study the complex linkages between behavioral plasticity and habitat distribution. We used a novel Markov model in a Bayesian framework to quantify the influence of behavioral state frequencies and environmental variables on transitions among landcover types through joint use of location and tri-axial accelerometer data. Data were collected from 56 greater white-fronted geese (*Anser albifrons frontalis*) across seven ecologically distinct winter regions over two years in midcontinent North America. We showed that goose decision-making varied across landcover types, ecoregions, and abiotic conditions, and was influenced by behavior. We found that time spent in specific behaviors explained variation in the probability of transitioning among habitats, revealing unique behavioral responses from geese among different habitats. Combining GPS and acceleration data allowed unique study of potential influences of an ongoing large-scale range shift in the wintering distribution of a migratory bird across midcontinent North America. We anticipate that behavioral adaptations among variable landscapes is a likely mechanism explaining goose use of highly variable ecosystems during winter in ways which optimize their persistence.

## Introduction

The demography and spatial distribution of animals is directly influenced by choices and consequences of resource use. Natural selection should favor animals that optimally use and select habitats that maximize fitness (i.e., reproductive success and survival^1^). Mechanistically, optimal resource use should manifest through observable modifications to habitat use patterns and adjustments to time allocated to specific behaviors (e.g., foraging, resting). These modifications should occur continuously through space and time as environmental or physiological conditions change, especially in heterogeneous environments^2,3^. Traditional investigations of animal resource use and selection have inferred that landcover types used in greater proportion than their availability on the landscape are selected for by animals because they are inherently beneficial to fitness^4^. The differences between resource use and selection in animal ecology have been well defined^4–6^, each providing unique but complementary information about biological and ecological requirements of animals in space and time. Habitat use is defined as “the way an animal uses the physical and biological resources in a habitat”^5^ and can include habitat used for a variety of activities (e.g., foraging, roosting, etc.). In tracking studies, habitat use is commonly measured as the amount of time spent, or number of relocations within, in a given landcover type (i.e., intensity of use). Habitat selection is a process whereby an animal makes choices regarding which landcover type to use given a known distribution of all landcover types available to it at varying spatiotemporal scales, and in a modeling framework, statistically compares characteristics of used and (assumed) non-used resources^5,6^. Investigating habitat use allows understanding of factors that influence the intensity of use in time, regardless of what is available to the animal on the landscape. For example, a landcover type (e.g., a specific agricultural crop) may be frequently used by an individual and would be deemed important in habitat use studies, but if that crop type commonly occurs within some availability threshold and there are many available resource units of the same crop that could be used but were not, it may be deemed as unimportant or “selected against” in habitat selection studies^6^, potentially negating its importance to the animal. Methods to accurately determine the habitat available to an animal at varying spatiotemporal scales often involve accepting assumptions that are unknown or untestable^4^, while investigations of habitat use requires fewer such assumptions because it relies only on known used landcover types. One limitation of interpreting resource selection strength and patterns is that the chosen availability threshold and number of random (i.e., non-used) locations can alter inferences made by resource selection studies^7,8^. Additionally, availability and selection of resources varies for individuals that use different ecosystems (e.g., arid vs. mesic), landscape configurations (e.g., homogeneous vs. heterogeneous) or experience different habitat conditions (i.e., a functional response in habitat use^9^). Although a primary goal of understanding resource selection, and in particular functional responses in habitat use, is to infer the behavioral mechanisms underlying selection patterns, these methods ignore potential changes in habitat use based on specific behavioral states (e.g., foraging) of individuals among and within habitats prior to determination of an appropriate method to estimate resource availability. Attempting to incorporate behavioral state into animal movement models has been the focus of many recent approaches that include step-selection and unsupervised or semi-supervised hidden Markov models^10,11^, yet many of these models only derive a latent behavioral state based on movement characteristics and not known, validated behaviors. Therefore, investigating the consequences of validated behavior on intensity of use in time may provide valuable information to inform resource selection in space. To more holistically address the implications and consequences of resource use and movement strategies by individuals, there is a need to incorporate the influence of animal behaviors in relation to habitat and environmental factors^12–14^.

Our ability to link fine-scale animal movement and behavior is the result of technological advancements in tracking devices, such as size and mass reductions, data collection and transmission capabilities, and battery capacity^15^, along with development of statistical methods to analyze high-frequency and highly autocorrelated data^16^. Through the advent of high-frequency GPS data, behavioral states can be derived through movement path segmentation methods based on speed, turning angle, step length, or a combination of these or more variables (e.g., hidden Markov models; [see Ref.^17^]). Most recently, tri-axial accelerometers have been used to either infer latent (i.e., hidden) behavioral states with unsupervised classification or directly estimate behavioral states using ground-truthed behavioral signatures with supervised classification^18,19^. Acceleration data (ACC) can be used to further understand how individual decision-making is manifested in individual behaviors, and thus allow researchers to link movement and behavioral processes to one another. The benefits of increasingly sophisticated technologies, like ACC, can be magnified when studying cryptic, highly mobile, or relatively inaccessible species, as the likelihood of encountering marked individuals in subsequent time intervals is low. Species that migrate long distances, such as Arctic-nesting geese, have been especially difficult for researchers to quantify spatiotemporal decision-making^20^. Despite these difficulties, it is imperative to understand movement and behavioral processes for future conservation planning and management of populations^14^, especially at the scale of wide-ranging, migratory species such as Arctic-nesting geese.

Globally, populations of many goose species have increased in recent decades due to increasing row-crop agriculture throughout migratory routes and wintering areas, which has reduced food resource limitations during the nonbreeding period^21^. The greater white-fronted goose (*Anser albifrons*) is a medium-sized goose species with a near-circumpolar distribution. In North America, the midcontinent population of greater white-fronted geese (*A. a. frontalis*, hereafter white-fronted geese) nests in both taiga and tundra ecosystems, from the Interior to the Arctic Coastal Plain of Alaska, USA, eastward across the Canadian Arctic to Hudson Bay^22^. Historically, white-fronted geese exclusively exploited the coastal marshes in Texas and Louisiana during winter. However, during the early twentieth century, white-fronted geese shifted their wintering range to inland habitat, largely due to their adaptable, generalist herbivore foraging strategy, declines in coastal marsh habitat, and a concomitant increase in acreage of grain agriculture, particularly rice^22^). Over the last two decades, white-fronted geese have shifted their main wintering distribution northeastward into the Mississippi Alluvial Valley (MAV) and now primarily winter in Arkansas, Louisiana, and Texas. However, the consequences of a shifting distribution on population dynamics, behaviors, resource use, and future management of white-fronted geese in midcontinent North America are largely unknown.

Continued loss of habitat through urbanization, shifting resource distributions (e.g., favorable crop types, water availability), and increasingly variable weather patterns driven by climate change may challenge the predictability of quality resources used by white-fronted geese during winter. Although winter is a critical period influencing goose fitness through cross-seasonal effects^23^, there is little information quantifying white-fronted goose habitat use or behavior on wintering areas. Thus, research investigating habitat use and behavioral decision-making in white-fronted geese may improve conservation planning and management strategies by informing regional habitat and bioenergetics models, and by examining mechanisms that may underly the observed distribution shift.

Our objective was to determine if known behavioral states influenced the intensity of habitat use and transition probability in time. We examined whether the probability of white-fronted geese transitioning among landcover types during winter was influenced by three behavioral states as well as relevant environmental variables. We hypothesized that the proportion of time spent in specific behaviors would explain variation in the probability of transitioning among landcover types. Additionally, we hypothesized that the influence of behavior on transitioning among landcover types would vary among ecologically distinct wintering regions due to differences in regional landscape composition. For example, geese in regions with abundant flooded agriculture such as the MAV, may not transition from agricultural landcover types to wetland landcover types when foraging decreases because flooded agriculture serves a similar functional role as natural wetlands^24^. Similarly, we examined the influence of two abiotic variables, time-of-day and temperature, on transitions among habitats. We hypothesized that transitions among landcover types would occur during diurnal rather than nocturnal hours^25^, and transitions to foraging habitats (i.e., agricultural grain crops) would occur early and late in the day, whereas transitions to wetland habitats would occur primarily during mid-day or evening periods. For temperature, we hypothesized that transitions to agricultural habitat would increase with decreasing daily temperature, as agricultural foods have higher caloric content than natural foods, and thus during colder temperatures, white-fronted geese should transition to agricultural foods to maximize energy gain^21,26^.

## Methods

### Goose capture and tracking

**Figure 1:**
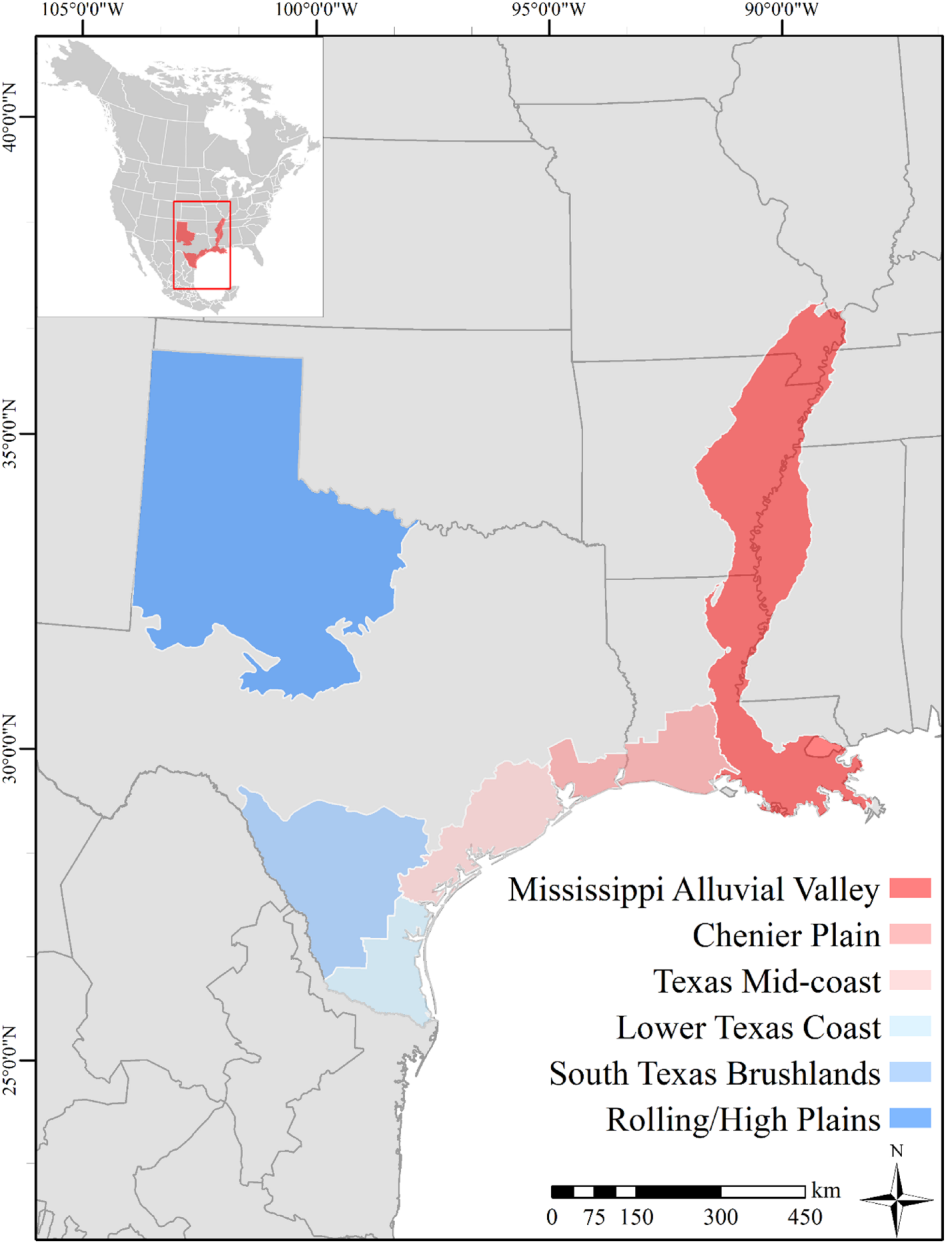
Primary wintering regions of the Midcontinent population of greater white-fronted goose (*Anser albifrons frontalis*) in North America (excluding regions in Mexico). Transmitters were deployed during winters 2016–2018 in the Chenier Plain (Louisiana), Lower Texas Coast, and Rolling/High Plains regions. Geese that made winter movements outside of these defined regions were classified as ‘Other’ regions. Map created using Esri ArcMap (version 10.3.1; www.esri.com).

We used rocket netting and leg snares to capture white-fronted geese in three regions in Texas (Rolling Plains, Lower Texas Coast, and South Texas Brushlands) and one region in Louisiana (Chenier Plain) from October to February 2016–2018 (Fig. 1). We determined age and sex of individuals by cloacal inversion, rectrices and other plumage characteristics^27,28^. We fit a solar powered GPS/ACC/Global System for Mobile communication (GSM) neckband tracking device (Cellular Tracking Technologies Versions BT3.0, BT3.5 and BT3.75; 44–54 g; Rio Grande, New Jersey, USA, and Ornitela OrniTrack-N38; 36 g; Vilnius, Lithuania), and an aluminum U.S. Geological Survey Bird Banding Laboratory metal leg band (Supplementary Fig. S1) on each bird. Geese were captured and tagged under USGS Bird Banding Permits #21314 and #23792, and Texas A&M University-Kingsville Institutional Animal Care and Use Committee #2015-09-01B. Captive geese were permitted under TAMUK IACUC #2018-01-11 and United States Fish and Wildlife Service Waterfowl Sale and Disposal permit #MB03808D-0. All applicable field methods were carried out in accordance with relevant guidelines and regulations. All animal handling protocols were approved by TAMUK IACUC committees and the USGS Bird Banding Laboratory. When multiple white-fronted geese were captured simultaneously, devices were only placed on adult females or adult males to eliminate the potential of placing devices on mated pairs, thus biasing independent data collection due to monogamous, long-term pair bonds in white-fronted geese. Location duty cycles were set to collect a GPS location every 30 min (i.e., 48/day) and location accuracy was 7.2 and 6.5 m for CTT and Ornitela devices, respectively. Data were uploaded once daily to respective online user interface websites when within areas of GSM coverage. When not in coverage areas, data were stored onboard the device until birds returned to coverage areas. All devices were equipped with a tri-axial ACC sensor which measured G-force (g; CTT devices) or millivolts (mV; Ornitela devices) at a fixed sampling scheme; CTT BT3.5 and Ornitela devices collected ACC data for a duration of 3 s every 6 min at 10 Hz, while BT3.0 devices collected data for a duration of 10 s every 6 min at 10 Hz. Generation BT3.0 devices were subsampled to match the sampling scheme of 3 s bursts before analyses. Ornitela units measured in mV were converted to G-force. We applied manufacturer- and tag-specific ACC calibration to all units, respectively, by collecting ACC data on each possible rotation for all axes when the device was stationary and applying the calibration to the raw ACC values (see Ref.^29^ for full calibration procedure). All devices recorded temperature in °C at each GPS fix. We censored GPS and ACC data from the time of release until individuals appeared to resume normal movement activity (i.e., roosting and foraging), as geese typically flew to the nearest wetland immediately after release where they remained without leaving while acclimating to wearing devices, which ranged from 1 to 7 days^30^. We defined the start of the winter period following a southward migratory movement from staging areas in Canada, without additional migratory movements southward below 40° 0′ 00″ N, or from the time of device deployment (minus device acclimation period) until geese made large northward migratory movements, or 28 February if geese remained in wintering areas.

### Land cover covariates

We used publicly available spatial landcover data sets (30-m resolution) in combination with remote sensing to create landscape layers using programs Esri ArcMap (version 10.3.1), Erdas Imagine, and Program R (version 3.5.2^31^). We used 2017 and 2018 National Agricultural Statistics Service Cropland Data Layer (CDL) data sets for agricultural crop types and freshwater wetlands, and the 2010 Coastal Change Analysis Program layer for saltwater and coastal wetland classifications^29,32^. Additionally, we used multi-spectral Landsat 8 Operational Land Imager satellite imagery, with principal component analysis on eight Landsat bands and a normalized difference vegetation index band, and unsupervised classification^33,34^ to accurately identify and create a spatial layer for peanut fields. We developed this layer for two regions with annual peanut agriculture (i.e., the Rolling/High Plains and South Texas Brushlands) using ground-truthed peanut fields, because the CDL layer did not identify this crop accurately based on our field observations during captures. We achieved > 90% accuracy of peanut identification for each image independently based on annual ground-truthed observations of peanut fields. Finally, we grouped like-habitat categories to reduce the total number of categories to eight: corn, grass/winter wheat, herbaceous wetlands, other grains (i.e., soybeans, sorghum, and peanuts), rice, woody wetlands, open water/unconsolidated shore and other (Supplementary Table S1). White-fronted geese used several ecologically distinct regions in both winters of our study (Fig. 1), where the landscape composition of specific landcover types varied. To account for regional variability, we added region ID as a categorical variable to all GPS locations. Regions included the MAV, Chenier Plain, Texas Mid-coast, Lower Texas Coast, South Texas Brushlands, Texas Rolling/High Plains, and Other (i.e., locations outside of these identified wintering regions; Fig. 1). We used regional shapefiles of Gulf Coast Joint Venture Initiative Areas (Laguna Madre [Lower Texas Coast], Texas Mid-coast, and Chenier Plain^35^), and Level III Ecoregions (Mississippi Alluvial Valley, Texas Rolling/High Plains, and South Texas Brushlands^36^) as boundaries to classify data into regions. Due to insufficient and incompatible spatial layers for Mexico, we limited analyses to locations within the US.

### Location and acceleration data collection

Remotely determining behaviors of individuals using ACC data is most accurately addressed by developing a training dataset of known behaviors linked with ACC measurements of those behaviors^18,37^. To develop a training dataset, we collected video footage of two domestic white-fronted geese in Texas, US, and 18 tagged wild Greenland white-fronted geese (*A. a. flavirostris*) fitted with the same device types and the same data collection scheme, in Wexford, Ireland and Hvanneyri, Iceland during winters 2017–2018. We supplemented wild recordings with behavioral recordings of captive white-fronted geese as a proxy for wild individuals due to difficulty filming wild geese in inclement weather and obstructed video footage, which is common in ACC literature^19,20,38,39^. To replicate devices placed on wild white-fronted geese and account for potential variation in ACC measurements between device brands, among device versions and individual geese, we deployed three versions of devices used in this study on captive white-fronted geese during filming sessions^38,40^. We attached tracking devices to captive geese one week prior to video collection to allow geese to adjust to wearing devices. We collected ACC measurements for 3 s bursts, at 1 min intervals, at 10 Hz. We constructed a 149 m^2^ enclosure in an agricultural field to imitate an environment that wild geese may encounter. We created two enclosure settings allowing captive geese to forage on sprouted winter wheat (~ 2–15 cm) or on a randomly dispersed mixture of grain seeds (corn, wheat, sorghum) to account for both ‘grazing’ of vascular vegetation and ‘gleaning’ of agricultural grains to imitate foraging in wild geese. We used Sony Handycam DCR-SR45 video cameras, matched internal camera clocks with a running Universal Coordinated Time clock, and verbally re-calibrated the current time every 2 min during video footage collection. We filmed 119.5 h of video footage, and matched behavior with recorded ACC measurements by pairing video and device timestamps for each device using JWatcher^41^ and Program R.

We characterized goose behaviors into four categories: ‘stationary’, ‘walk’, and ‘foraging’ from ground-truthed video footage, and ‘flight’ from visual inspection of the ACC data and consecutive GPS tracks during migration where device-measured speed remained > 4.63 km/h (based on a natural break in the speed density distribution of all GPS locations). Each ACC burst was classified as only one behavior (i.e., a goose that was walking as it foraged was classified as ‘foraging’). We combined wild goose behaviors and captive goose behaviors after identifying minimal differences in ACC burst summary statistics^29^ for ‘stationary’ and ‘walk’ behaviors. We used ‘graze’ behaviors only from wild geese because of low sample size for captive geese and slight differences in ACC summary statistics between captive and wild geese for this behavior. ‘Glean’ foraging behavior was only classified from captive geese. We then combined ‘graze’ and ‘glean’ behaviors into an overall ‘foraging’ behavior to account for variation in foraging behavior of wild geese, and because machine learning models could not accurately distinguish between the two foraging modes^40^. We randomly subsampled all behaviors to 150 bursts if our dataset contained at least that many bursts to reduce the risk of artificially increasing prediction accuracy^20^. We determined there were insufficient differences in ACC signatures between CTT BT3.0 and BT3.5 versions by visual comparison of signatures and summary statistics, and merged all bursts into an overall CTT-specific training data set, and retained CTT- and Ornitela-specific training data sets to account for brand-specific ACC measurement schemes. The final training data sets consisted of 150 stationary, 150 walking, 118 foraging, and 150 flying bursts (CTT), and 150 stationary, 75 walking, 120 foraging, and 150 flying bursts (Ornitela).

We used the training data sets to predict behaviors of tagged, wild white-fronted geese during winter with temporally aligned GPS and ACC data. We used a suite of supervised machine-learning algorithms and selected the algorithm with greatest prediction accuracy based on an 80% training, 20% testing sample approach. We tested random forest, support vector machines, K-nearest neighbors, classification and regression trees, and linear discriminant analysis, all with cross validation in Program R^18,29,42^. We evaluated models using three metrics defined in Ref.^42^: (1) overall classification accuracy as the percent of classifications in the test data set that were predicted correctly, (2) precision of assignment, the probability that an assigned behavior in the test data set was correct, and (3) model recall, the probability that a sample with a specific behavior in the test data set was correctly classified as that behavior by the model. Random forests provided the highest overall classification accuracy (95.6% for CTT and 96.0% for Ornitela), as well as high precision and recall for each behavior (CTT range 93.1–99.3, Ornitela range 88.9–100.0%), and therefore we labeled behaviors from wild goose ACC data using the random forests.

### Habitat transition model

Our habitat-transition model required temporally matched GPS and ACC datasets. Therefore, we subset all GPS locations to match the time-series of ACC data per individual because devices typically acquired GPS data longer than ACC data before device failure or individual mortality. For each GPS location, we extracted the landcover type and wintering region from spatial layers and retained temperature recorded from the device. To link classified ACC behaviors to GPS locations, we matched ACC timestamps between two GPS locations with the previous GPS timestamp. That is, all ACC bursts between two GPS locations were assigned backward to the previous GPS location. In this way, an individual’s first location is collected in GPS landcover type A, ACC data are collected in 5 bursts, their behaviors are classified and assigned to the first GPS location A and associated landcover type, followed by collection of GPS location B, in which the subsequent 5 ACC bursts are associated to GPS location/landcover type B. In the case of missing GPS locations, we matched ACC bursts to the previous GPS location only if the ACC timestamps were within 60 min of the GPS timestamp, and ACC bursts occurring greater than 60 min after GPS acquisition were removed until the next GPS fix. To account for temporal variation in habitat-behavior relationships, we calculated two continuous covariates representing time-of-day based on the local time associated with the timestamp of each GPS location for each individual. The variable cos(Diel) represented diurnal (negative values) and nocturnal (positive values) periods, and sin(Time) represented midnight until 11:59 a.m. (positive values) and noon until the following 11:59 p.m. (negative values), where high and low values ranged continuously between 1 and − 1^43^. Our temporally matched time series of GPS and ACC data yielded 53,502 GPS locations linked with 300,348 ACC bursts across both winters.

We used a Bayesian Markov model with Pólya-Gamma sampling following^43^), [cf. Refs.^44,45^] to determine how transitions between landcover types were influenced by behavior, temperature, time-of-day, and wintering region. The proportion of time spent foraging, walking, and stationary between each successive GPS fix was included as a covariate; flight was not included to reduce multicollinearity due to behavior proportions summing to one. Markov models account for non-independence among observations by assuming that the current state (i.e., landcover type) is dependent upon the previous state, and allow the determination of covariate influences on the probability of transitioning among states through a logistic link function. The transition probability from habitat *i* to habitat *j* at time *t* for individual *n* is modeled with multinomial logistic regression:

$$\begin{aligned} & logit\left( {p_{nijt} } \right) = log\left( {\frac{{p_{nijt} }}{{p_{niJt} }}} \right) = \mathop \sum \limits_{{r \in {\mathcal{R}}_{j} }} \beta_{0jr} I\left( {Region_{nt} = r} \right) + \beta_{1j} {\text{cos}}\left( {Diel_{nt} } \right) \\ & \quad + \beta_{2j} {\text{sin}}\left( {Time_{nt} } \right) + \beta_{3ij} Forage_{nt} + \beta_{4ij} Walk_{nt} + \beta_{5ij} Stationary_{nt} + \beta_{6ij} Temperature_{nt} , \\ \end{aligned}$$

where ${\mathcal{R}}_{j}$ is the set of wintering regions $r$ where habitat $j$ occurs, $Regio{n}_{rnt}$ indicated wintering region $r$, and $\mathrm{cos}\left({Diel}_{nt}\right)$ and $\mathrm{sin}({Time}_{nt})$ were temporal covariates (described above) for habitat *j*. Quantities ${Forage}_{nt}, {Walk}_{nt},\mathrm{ and }{Stationary}_{nt}$ were the scaled (mean = 0, standard deviation = 1) proportion of time spent in each behavior between transitions from habitat *i* to habitat *j*, and ${Temperature}_{nt}$ was scaled ambient temperature (°C) for transitions from habitat *i* to habitat *j*. All coefficients for transitions to the baseline habitat $J$ were set to 0 (i.e., ${\beta }_{0Jr}$ for all $r$, ${\beta }_{1J}$, ${\beta }_{2J}$, ${\beta }_{3iJ}$, ${\beta }_{4iJ}$, ${\beta }_{5iJ}$,${\beta }_{6iJ}$, for all $i$). We set the baseline habitat $J$ as open water/unconsolidated shore because this habitat is used primarily for both nocturnal roosting and diurnal loafing, included all behaviors, and transitions to all other landcover types were frequent in each region.

The prior for the set of winter region intercepts for each habitat was:

$${\beta }_{0jr}\sim N({\beta }_{0j},{\sigma }_{0jr}^{2}),$$

for $r\in {\mathcal{R}}_{j}$, ${\beta }_{0j}$ was the mean intercept, and ${\sigma }_{0jr}^{2}$ was set to 100. For ${\beta }_{0j}$, a vague prior mean 0 and σ^2^ = 100 was used with an assumed normal distribution.

The Markov model was executed within a Bayesian framework to robustly quantify uncertainty. The Markov model assumed that data were collected at regular time intervals for both GPS (30 min) and ACC (6 min), however imperfect collection by devices created irregular data sets. Therefore, we subsampled GPS locations and constrained time series data to sequences where GPS locations missing > 120 min intervals (i.e., 4 locations) were separated into sequences of regular time series data for each individual^46^. We extended^43^ by including a mix of both transition-specific effects (i.e., behaviors, temperature) and habitat-specific effects (i.e., wintering region, cos(Diel), and sin(Time)), where transition-specific effects were allowed to vary for a current habitat state, while habitat-specific effects were not. We included a mix of coefficients because initial model runs indicated that some effects were similar regardless of the current habitat (i.e., were habitat- and not transition-specific decisions). We also incorporated a model feature to exclude estimation of transitions that did not occur either within the dataset as a whole or within each specific wintering region because landcover types varied among them by setting those specific transition probabilities to zero. We centered and standardized all behavior and temperature covariates, sampled 50,000 iterations from the model posterior using one chain, and discarded the first 10,000 iterations as burn-in. We assessed model convergence by evaluating trace plots and setting random initial values, examined autocorrelation plots, and Geweke diagnostics using the R package ‘coda’^47–49^.

## Results

### Tracking, habitat use and behaviors

We deployed 127 devices (118 CTT and 9 Ornitela) during winters 2016–2018, but only 56 devices (49 CTT and 7 Ornitela) provided temporally-matched GPS and ACC data for at least 1 day before device failure or bird mortality. On average, devices provided 27.5 (± SE 3.5) days of data during winter, ranging from 1 to 132 days per device (Supplementary Fig. S2). In this analysis, we included 47 devices deployed in Texas (*n* = 28 in 2016–2017; *n* = 19 in 2017–2018) and 9 devices in Louisiana (*n* = 9 in 2016–2017) on after-hatch-year (52 female, 4 male) white-fronted geese (Table 1). Devices performed better in 2016–2017 (57,084 total locations; $\overline{x }$ = 43.0 ± 0.46 locations/device/day for a 127 day winter period, range = 24–49) than in 2017–2018 (41,456 total locations; $\overline{x }$ = 29.6 ± 0.50 locations/device/day for a 137 day period, range = 14–43). Information regarding tracking duration per individual, habitat use frequency, time-activity budgets, and proportion of time spent in each activity per habitat can be found in the Supplementary Information.

**Table 1:** Capture details for 56 greater white-fronted geese (*Anser albifrons frontalis*) including capture year, month, state, and region, individual sex, and tracking device information for geese captured during winters 2016–2017 and 2017–2018.

Year	Month	State	Region	*n*	Sex
2016	February	TX	Rolling Plains	2	F
	October	LA	Chenier Plain	3	2 F, 1 M
		TX	Lower Texas Coast	3	1 F, 2 M
	November	LA	Chenier Plain	6	F
		TX	Lower Texas Coast	5	F
	December	TX	South Texas Brushlands	3	F
2017	January	TX	Lower Texas Coast	4	3 F, 1 M
	February	TX	Lower Texas Coast	2	F
		TX	Rolling Plains	9	F
	November	TX	Lower Texas Coast	12	F
		TX	Lower Texas Coast	2	F
2018	January	TX	Rolling Plains	5	F

### Habitat-specific effects

**Figure 2:**
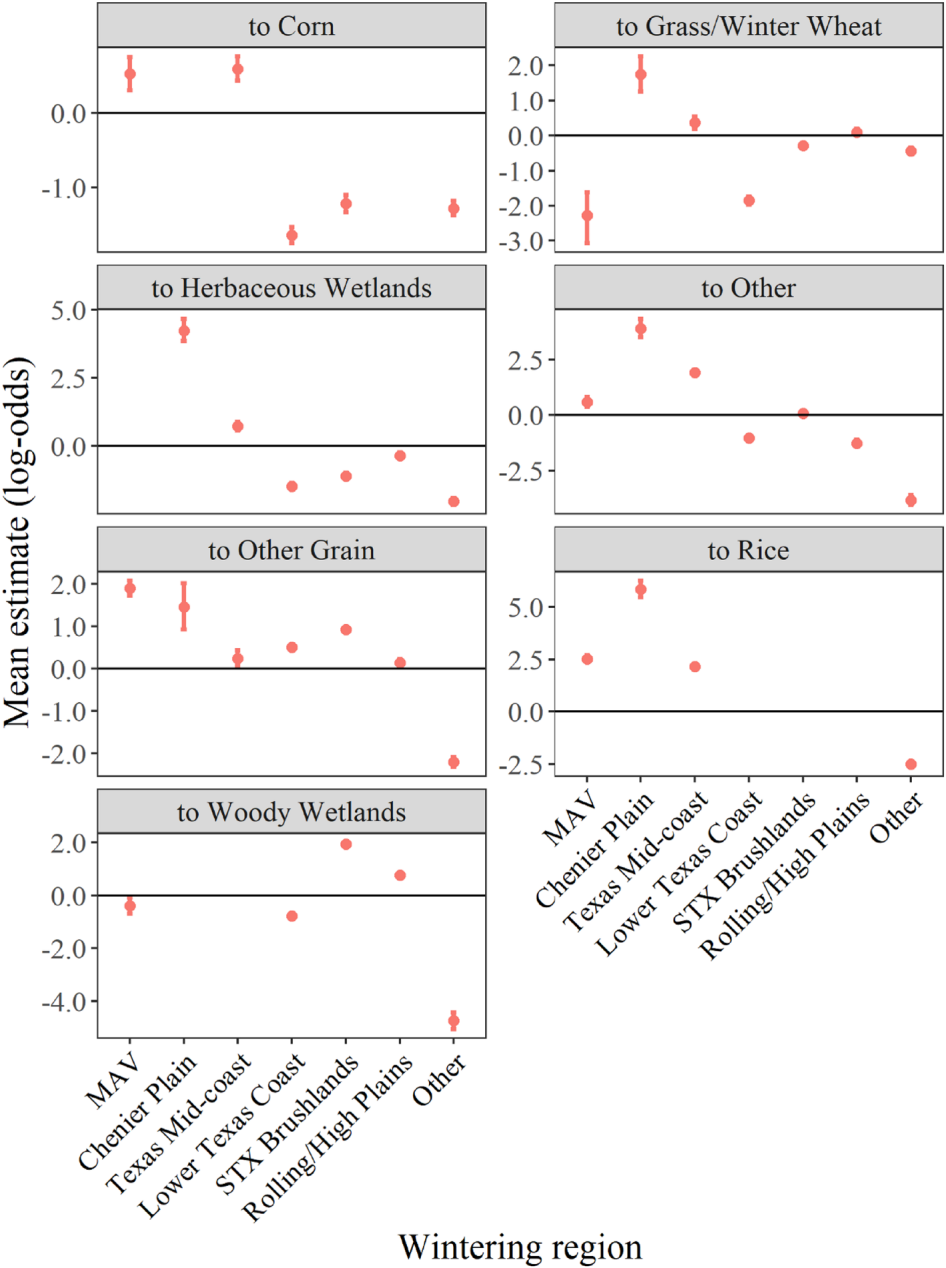
Mean effect on habitat-specific transition probability (± 95% credible intervals; log-odds scale) to each of seven landcover types for the specific wintering region selected by 56 greater white-fronted geese during winters 2016–2017 and 2017–2018. Note varying scales of the y-axis on each panel to show increased detail due to small credible intervals. Also, effects were not estimated for transitions that were not observed; for example, no estimate is reported for transitions to corn in Chenier Plains in the upper left panel.

Habitat-specific effects determined the probability of transitioning to a landcover type from all other previous landcover types combined (i.e., from any landcover type), given the covariate, and are relative to the probability of transitioning to the baseline habitat, open water/unconsolidated shore. Probabilities of transitioning to specific landcover types were strongly influenced by wintering region (Fig. 2). For example, the effect of transitioning to corn was positive (i.e., more probable) for the MAV and Texas Mid-coast, and negative (i.e., less probable) for all other regions indicating that corn is more likely to be used in these regions than others. Similarly, the probability of transitioning to woody wetlands was positive only for the South Texas Brushlands and the Rolling/High Plains, and negative for all other regions highlighting the importance of woody wetlands in those regions. Transition probabilities were negative for all habitats in the Other region, indicating that all transitions were less probable than transitions to the reference category, open water/unconsolidated.

**Figure 3:**
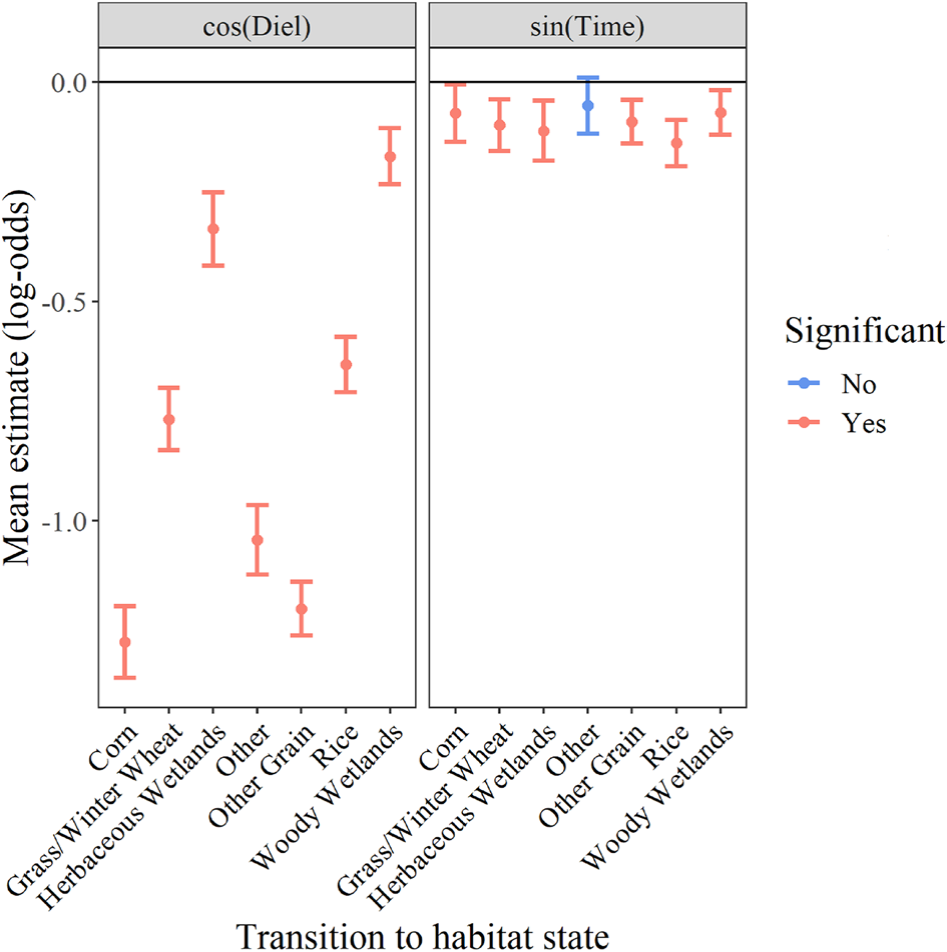
Mean effect on habitat-specific transition probability (± 95% credible intervals; log-odds scale) to each of seven landcover types for time-of-day covariates cos(Diel) and sin(Time). The variable cos(Diel) represents nocturnal and diurnal time periods, while sin(Time) represents the first half (12:00 a.m.–11:59 a.m.) and second half of the day (12:00 p.m.–11:59 p.m.).

Transition probabilities for sin(Time) were relatively consistent among all landcover types, but were more variable for cos(Diel) (Fig. 3). All transitions for sin(Time) were negative, suggesting transitions were more probable during the second half of the day. Credible intervals for all transitions in sin(Time) overlapped one another, indicating a similar effect across all habitat-specific transitions. Probabilities for cos(Diel) were all significantly negative, and effects were stronger than for sin(Time), indicating that habitat transitions are more probable during diurnal hours of the day than nocturnal hours. Transitions to foraging habitats had higher probabilities of occurring during diurnal hours than transitions to herbaceous wetlands and woody wetlands (Fig. 3). Effects closer to 0, while still negative, indicate that transitions to wetland habitats were more probable earlier and later during diurnal hours (remaining in and transitioning to roosting habitat) than transitions to foraging habitat.

### Transition-specific effects

**Figure 4:**
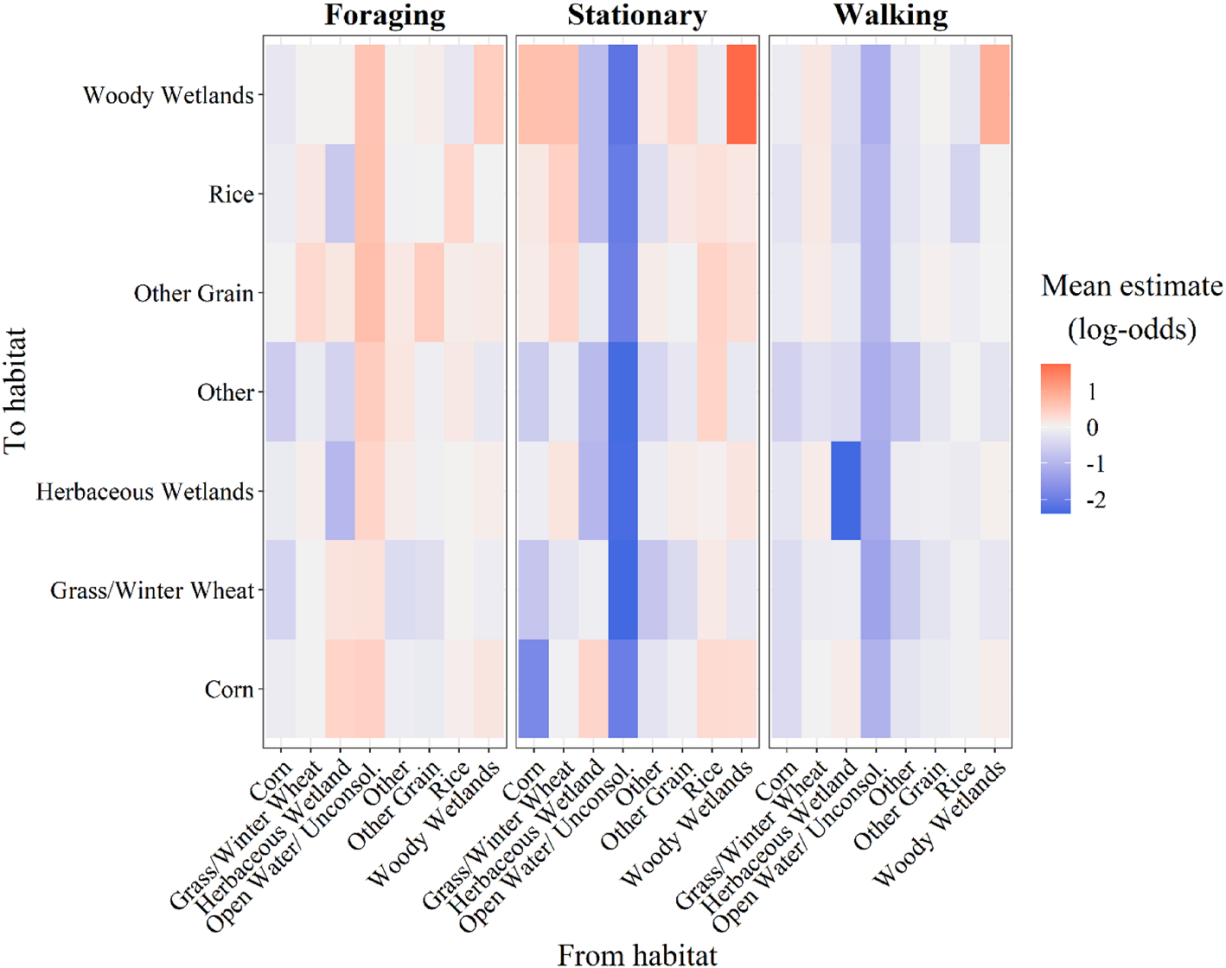
Mean effect on transition-specific probability (log-odds scale) of transitioning between all combinations of landcover types for the proportion of time spent performing each of three behaviors including foraging, stationary, and walking between each GPS location. Positive estimates reflect an increased probability of transitioning from a habitat to a habitat given an increase in the proportion of time spent in a specific behavior, and negative estimates reflect an increase in probability of transition given a decrease in the proportion of time spent in a specific behavior.

**Figure 5:**
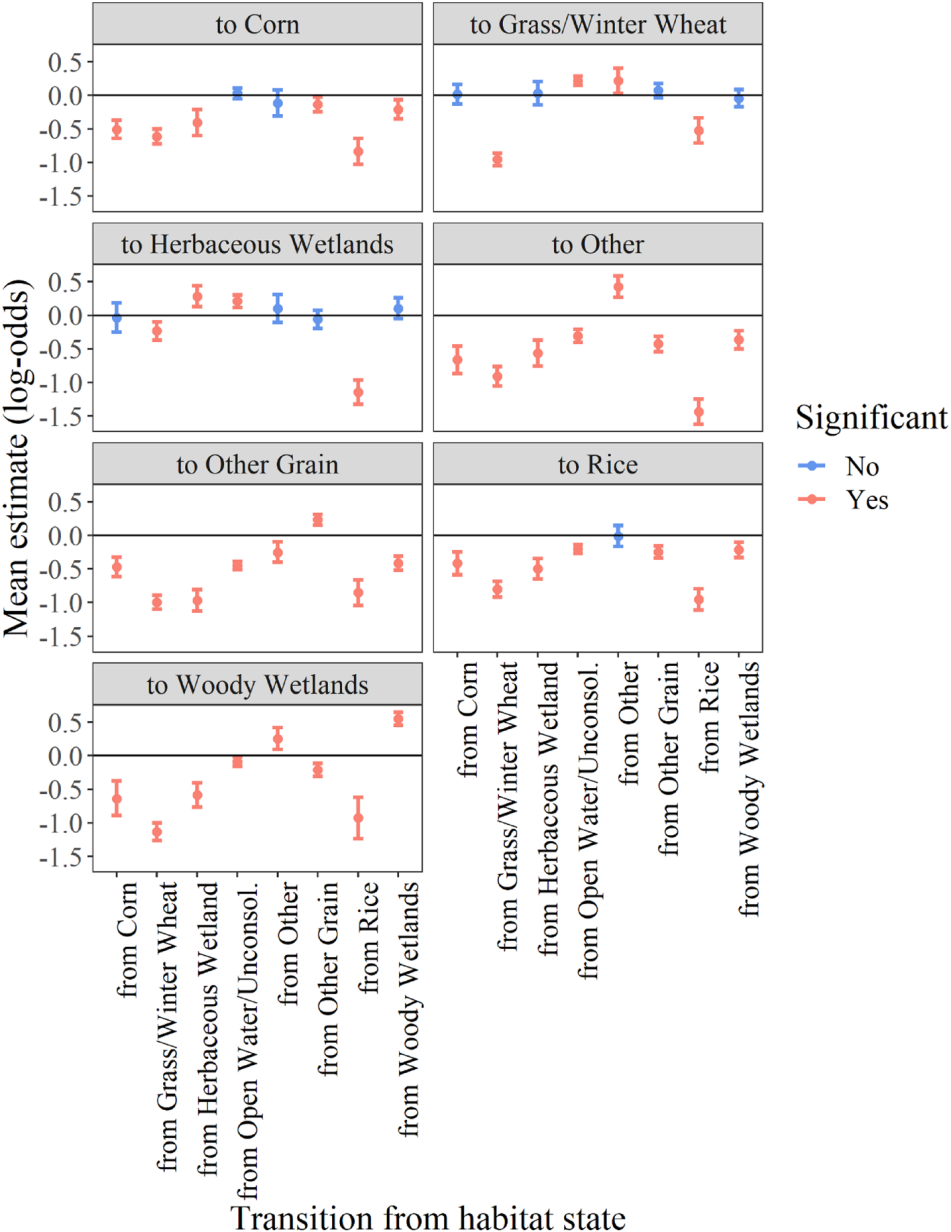
Mean effect on transition-specific probability (± 95% credible intervals; log-odds scale) of transitioning between all combinations of landcover types for ambient temperature (°C) derived from tracking devices. Negative estimates reflect transitions that were more probable at lower than average temperature, and positive estimates at higher than average temperature.

Transition-specific effects determined the probability of transitioning to a landcover type from all other previous landcover types independently (i.e., each combination of landcover types), given the covariate of interest, relative to the probability of transitioning to the baseline habitat, open water/unconsolidated shore. We observed transition-specific effects of both behavior and temperature on the probability of transitioning among landcover types (Figs. 4, 5). Transition probabilities varied considerably based on the starting landcover type, transitioning to landcover type, as well as the behavior, revealing complex decision-making strategies. However, some patterns were apparent among all behaviors. For example, the probability of transitioning to all other landcover types from open water/unconsolidated increased as the proportion of time spent stationary and walking decreased, and as foraging increased (Fig. 4), indicating that geese primarily use open water/unconsolidated habitat for stationary or walking behaviors and not foraging, which is consistent with roosting behavior. When time spent foraging increased in open water/unconsolidated, geese were more likely to transition to croplands (Fig. 4). For instance, the probability of geese transitioning to other grain increased as time spent foraging increased, suggesting geese used other grain largely for foraging. Geese transitioned to woody wetlands from other landcover types and remained in woody wetlands when time spent stationary increased, suggesting this wetland type functions as nocturnal roosting and/or diurnal loafing habitat (Fig. 4). The influence of behavior on transitioning to herbaceous wetlands, however, was more dynamic, as the proportion of time spent in each behavior from all other landcover types (except herbaceous wetland and open water/unconsolidated) did not affect the probability of transitioning to herbaceous wetlands (Fig. 4).

Temperatures experienced by white-fronted geese averaged across all regions were nearly 5 °C warmer during winter 2016–2017 (19.4 ± 0.04 SE °C) than 2017–2018 (14.9 ± 0.06 °C). During winter 2016–2017, < 0.001% of temperature measurements collected at GPS locations were below 0 °C, and < 0.04% during winter 2017–2018. Lower temperatures increased the probability of transitioning to most (82.2%) landcover types (Fig. 5). This relationship was especially apparent for transitions to grains, such as corn, other grain, and rice. For example, probabilities of transitions to rice from all habitats except other increased as temperature decreased. Lower temperatures also increased the probability of remaining in agricultural landcover types (i.e., corn, rice, grass/winter wheat), whereas higher temperatures increased the probability of remaining in wetland landcover types (i.e., woody wetlands, herbaceous wetlands). However, the influence of temperature on transition probabilities to all foraging habitats (i.e., agriculture) was not consistent, as transitions to grass/winter wheat were more variable than other foraging landcover types (Fig. 5). The effect of temperature on the probability of transitioning varied between wetland types as well. For example, most of the probabilities of transitioning to herbaceous wetlands were relatively small and less affected by temperature, whereas transitions to woody wetlands were more probable at lower temperatures for all landcover types except other and woody wetlands, suggesting that transitions to woody wetlands are much more probable at lower temperatures than transitions to herbaceous wetlands.

## Discussion

We used a novel application of joint GPS and ACC data to show that specific behavioral states and their frequency influenced decisions on intensity of habitat use by migratory animals across a seasonal geographic range. Greater white-fronted geese made non-random habitat transition decisions based on dynamic behaviors, which were both behavior and habitat-dependent, although some of which were universal among all landcover types given specific behaviors performed. Additionally, we found that decision-making strategies were influenced by geographic and environmental factors. The particular ecoregion that individuals occupied, time-of-day, and temperature all revealed either habitat-specific or transition-specific effects on habitat use transitions. We provided evidence that white-fronted geese use and transition among different landcover types when their behaviors change, and therefore use different landcover types for different purposes even when landcover types were generally similar. For example, transitions to agricultural crops used for foraging such as corn and other grains were influenced differently by behavior, whereby transitions to other grains increased when foraging increased in all other landcover types, but transitions to corn increased when foraging in wetland landcover types increased.

Our ability to spatiotemporally link GPS and ACC data that were classified using known behavioral signatures greatly strengthened our inferences of individual decision-making. Contemporary approaches to understand individual decision-making in relation to resource use, movements, and behaviors such as resource-, step-, and path-selection functions and state-space modeling (e.g., hidden Markov models [HMM]) have rarely included validated, labeled behaviors to improve inference. Commonly used resource selection analyses do not include the influence of specific, supervised behavioral states, but more recently have begun to include coarse movement states (e.g., active and inactive)^50–52^, or have estimated resource selection functions based on unvalidated movement states derived from movement trajectories to investigate behavioral influences on resource selection^53,54^. Additionally, while resource selection studies often include space and time^55^, few studies have attempted to understand fine-scale spatiotemporal decision-making patterns of habitat use among ecologically-distinct regions across a seasonal range of a broadly distributed migratory species. Because of this, our objective was to determine if known behavioral states influenced the intensity of habitat use and transition probability in time, and not to quantity behavioral influences on habitat selection. Our model allowed us to determine that behavior influenced decisions that individuals made regarding the type and the intensity of use of landcover types without defining an availability distribution, freeing our analyses from assumptions which may be difficult to satisfy in a resource selection function framework (e.g., individuals have access to all defined units deemed available to them^4^). Incorporating individual decision-making through known behavioral influences on habitat use into habitat selection models could be informative and the subject of future studies. The unsupervised and semi-supervised HMM frameworks use characteristics of movement trajectories to estimate an a priori researcher-selected number of latent movement states, which are subsequently classified into expected behaviors (i.e., unvalidated) based on expert interpretation to determine biological behaviors (e.g., foraging, resting) constituting underlying movement states^17^. The introduction of observer subjectivity in the number of states assumed to be present in the movement trajectory^56^, latent behavioral assignment, and inability of specific behaviors to be detected introduces bias into inferences and necessitates the need for validated behaviors to best inform animal movement models^57^. By modeling both GPS and ACC data in a joint framework, our model incorporated validated, biologically meaningful, supervised behavioral states which allowed for more detailed interpretation of effects and robust inference.

Consistent with our hypothesis, white-fronted geese exhibited differential probabilities of habitat-specific transitions based on their wintering region. In instances where landcover types used by geese are generally similar between regions (Supplementary Information), such as MAV and Chenier Plain, transition probabilities showed differential use of the same landcover types. For example, transition probability to rice exhibited a similar sign but differing effect strength between the two regions, whereas grass/winter wheat showed completely opposite effects (Fig. 2). These differences indicate adaptability in regional habitat use decision-making strategies by white-fronted geese based on individual needs and regional habitat resources^58^. White-fronted geese make frequent large-scale movements among ecologically distinct regions both within and between wintering periods^30^, and the ability to make region-specific decisions allows them to exploit an array of diverse ecosystems successfully. Our habitat-transition model is flexible in the ability to ignore transitions which cannot occur due to the absence of specific landcover types from the region. Understanding regional habitat use and decision-making by white-fronted geese is essential for continued management during the winter period, especially during a continued distribution shift.

Our model provided reliable estimates of temperature effects on habitat use transitions in white-fronted geese supported by previous studies of goose behavior and temperature relationships. White-fronted geese had higher probabilities of transitioning to agricultural grain crops at lower temperatures and to wetland habitats at higher temperatures, consistent with our hypothesis. Geese primarily decrease movements or cease specific behaviors such as foraging below temperature thresholds which incur net energy deficits^26,59^. However, white-fronted geese in this study rarely endured temperatures below 0 °C, and even more rarely below the thermoregulatory lower critical limit presumed for geese (e.g., − 6 °C for Canada geese [*Branta canadensis*]^60^. Ely^26^ showed that white-fronted geese did not drastically change behavior until temperatures were well below freezing (i.e., − 12 °C), when they spent nearly 70% of daylight hours roosting. Therefore, transitions among landcover types were more probable at cooler temperatures likely because geese required increased energy intake. When temperatures were warmer than average, geese typically transitioned only to wetland, other, or grass/winter wheat landcover types. Wetland and other habitats are primarily used for resting as geese were stationary in these landcover types (Supplementary Fig. S4). Grass/winter wheat contains less digestible energy than waste agricultural grains that are needed during colder temperatures, and geese fed less in grass/winter wheat than in agricultural grains (Supplementary Fig. S3)^21^. Jónsson and Afton^61^ found that both lesser snow geese (*Anser caerulescens caerulescens*) and Ross’s geese (*A. rossii*) in Louisiana increased time spent feeding when temperatures decreased during winter, and decreased time spent feeding and locomoting when temperatures increased.

White-fronted geese exhibited predictable responses in habitat transitions for both time-of-day effects, with high probability of transitioning among habitats during diurnal hours, and transitions to foraging habitats, which were generally similar in the first and second halves of the day. White-fronted geese are generally central-place foragers, where nocturnal roosting and diurnal loafing occur on wetlands, which serve as the center, and radiate to agricultural foraging locations during crepuscular periods^26^. The covariate sin(Time) provided evidence that transitions generally occurred during the second half of the day, but transition probabilities were similar among all landcover types, indicating no major differences among landcover types used in the first half or the second half of the day. Geese are largely active during the diurnal period, but can show increased activity during nocturnal periods of bright moonlight when high diurnal disturbance necessitates nocturnal foraging, or during migration^25^. The covariate cos(Diel) showed strong effects of transitions to all landcover types that were more probable during diurnal rather than nocturnal periods, consistent with our predictions. However, this effect varied among landcover types where wetland habitats had a lower probability than agricultural habitats, indicating that these transitions were more probable earlier and later within the diurnal period corresponding to crepuscular periods. Our model allowed time-of-day effects to be habitat-specific and not dependent on the ‘from habitat’ state, because we hypothesized that white-fronted geese made similar habitat transition decisions early and late in the day, and between nocturnal and diurnal periods.

We anticipate increased precision in habitat transition probabilities when including additional classified behaviors. Increasing the number of behaviors could be achieved through increasing the frequency or duration of ACC measurements to construct more detailed behavioral signatures. However, this should be dependent on actual animal behavior durations and device storage and transmission capabilities^18^. Accelerometers are more accurate at predicting behaviors with greater head movement (e.g., foraging) when placed on the neck than backpack-style devices^20,62^. Although we did not account for other factors that may influence behavioral state durations in geese, such as social hierarchy in flocks (e.g., family group with presence of goslings, paired or unpaired geese), or flock size, our goal was to quantify individual decision-making strategies made by geese relative to their behavior regardless of these factors to encompass strategies of all adult individuals of varying status and quality within the population. Increasing the GPS location collection frequency would increase precision, in addition to a greater number of identified behaviors, could more finely assess habitat transitions. Finally, incorporating additional covariates such as social status may yield beneficial information as to habitat transition rates and the role of social hierarchy in animal population interactions with the environment.

How animals use their environment is dependent upon perceived fitness benefits, requiring frequent decision-making. Advances in animal tracking technologies have rapidly improved our understanding of individual decision-making across animal ecology. Highly accurate GPS locations combined with near continuous behavioral identification alleviates common constraints in understanding interactions among habitat use, behavior, and decision-making (e.g., time- and visibility-limitations, observer bias, sampling behavior in representative landcover types, diurnal vs. nocturnal behavior identification, etc.^63^. Combining habitat use and behavior, tracking technologies and novel statistical tools allows for unprecedented insight into how animals make decisions about transitioning within and among landcover types. In a conservation framework, these advances may be used to make predictions or simulations of how animals will use and behave in specific areas by including both region-specific landcover types and abiotic interactions with habitat use and behaviors. Additionally, they may aid in determining how animals respond to habitat loss or replacement, restored habitat, or how future conservation scenarios may influence use and decision-making by animals.

## Supplementary Information

Supplementary Information.

## Data Availability

The datasets generated during and/or analyzed during the current study are available from the corresponding author on reasonable request.

## References

[1] Houston AI, McNamara JM (2014). Foraging currencies, metabolism, and behavioural routines. J. Anim. Ecol..

[2] Nathan R (2008). A movement ecology paradigm for unifying organismal movement research. Proc. Natl. Acad. Sci. U.S.A..

[3] Owen-Smith N, Fryxell JM, Merrill EH (2010). Foraging theory upscaled: The behavioural ecology of herbivore movement. Philos. Trans. R. Soc. Lond. B. Biol. Sci..

[4] Manly, B. F. L. et al. *Resource Selection by Animals: Statistical Design and Analysis for Field Studies* (Springer Science & Business Media, 2007).

[5] Krausman PR (1999). Some basic principles of habitat use. Graz. Behav. Livest. Wildl..

[6] Lele SR, Merrill EH, Keim J, Boyce MS (2013). Selection, use, choice, and occupancy: Clarifying concepts in resource selection studies. J. Anim. Ecol..

[7] Beyer HL (2010). The interpretation of habitat preference metrics under use-availability designs. Philos. Trans. R. Soc. Lond. B Biol. Sci..

[8] Northrup JM, Hooten MB, Anderson CR, Wittemyer G (2013). Practical guidance on characterizing availability in resource selection functions under a use-availability design. Ecology.

[9] Mysterud A, Ims RA (1998). Functional response in habitat use: Availability influences relative use in trade-off situations. Ecology.

[10] Langrock R (2012). Flexible and practical modeling of animal telemetry data: Hidden Markov models and extensions. Ecology.

[11] Thurfjell H, Ciuti S, Boyce RA (2014). Applications of step-selection functions in ecology and conservation. Move. Ecol..

[12] Roever CL, Beyer HL, Chase MJ, van Aarde RJ (2013). The pitfalls of ignoring behaviour when quantifying habitat selection. Divers. Distrib..

[13] Shaw AK (2020). Causes and consequences of individual variation in animal movement. Move. Ecol..

[14] Wittemyer G, Northrup JM, Bastille-Rousseau G (2019). Behavioural valuation of landscapes using movement data. Philos. Trans. R. Soc. Lond. B Biol. Sci..

[15] Hebblewhite M, Haydon DT (2010). Distinguishing technology from biology: A critical review of the use of GPS telemetry data in ecology. Philos. Trans. R. Soc. Lond. B Biol. Sci..

[16] Smouse PE (2010). Stochastic modelling of animal movement. Philos. Trans. R. Soc. Lond. B. Biol. Sci..

[17] Edelhoff H, Signer J, Balkenhol N (2016). Path segmentation for beginners: An overview of current methods for detecting changes in animal movement patterns. Move. Ecol..

[18] Nathan R (2012). Using tri-axial acceleration data to identify behavioral modes of free-ranging animals: General concepts and tools illustrated for griffon vultures. J. Exp. Biol..

[19] Wang G (2019). Machine learning for inferring animal behavior from location and movement data. EcoI.

[20] Weegman MD (2017). Using accelerometry to compare costs of extended migration in an arctic herbivore. Curr. Zool..

[21] Fox AD, Abraham KF (2017). Why geese benefit from the transition from natural vegetation to agriculture. Ambio.

[22] Baldassarre, G. A. *Ducks, Geese, And Swans of North America*. (Wildlife Management Institute, Johns Hopkins University Press 2014).

[23] Sedinger JS, Alisauskas RT (2014). Cross-seasonal effects and the dynamics of waterfowl populations. Wildfowl Spec. Issue.

[24] Elphick CS (2000). Functional equivalency between rice fields and seminatural wetland habitats. Conserv. Biol..

[25] McNeil R, Drapeau P, Goss-Custard JD (1992). The occurrence and adaptive significance of nocturnal habits in waterfowl. Biol. Rev..

[26] Ely CR (1992). Time allocation by greater white-fronted geese: Influence of diet, energy reserves, and predation. Condor.

[27] Dzubin, A. X., & Cooch, E. G. *Measurements of Geese: General Field Methods*. (California Waterfowl Association, 1992).

[28] Hochbaum HA (1942). Sex and age determination of waterfowl by cloacal examination. Trans. N. Am. Wildl. Nat. Resour. Conf..

[29] Cunningham, S. A. Decision-making and demography of greater white-fronted geese. Thesis, University of Missouri-Columbia, Columbia, MO (2019).

[30] VonBank JA (2021). Winter fidelity, movements, and energy expenditure of Midcontinent greater white-fronted geese. Move. Ecol..

[31] R Core Team. R: A Language and Environment for Statistical Computing. https://www.R-project.org/ (2017).

[32] National Oceanic and Atmospheric Administration, Office for Coastal Management. Coastal Change Analysis Program (C-CAP) Regional Land Cover. Charleston, SC: NOAA Office for Coastal Management. www.coast.noaa.gov/ccapftp (accessed 01 July 2017 ).

[33] Campbell, J. B. & Wynne, R. H. *Introduction to Remote Sensing*, 5th ed. (The Guildford Press, 2011).

[34] Schowengerdt, R. A. *Remote Sensing: Models and Methods for Image Processing* (Elsevier, 2006).

[35] Brasher, M. G., James, J. D., & Wilson, B. C. *Gulf Coast Joint Venture Priority Waterfowl Science Needs*. (Gulf Coast Joint Venture, 2012).

[36] U.S. Environmental Protection Agency. Level III ecoregions of the continental United States: Corvallis, Oregon, U.S. EPA—National Health and Environmental Effects Research Laboratory, map scale 1:7,500,000. https://www.epa.gov/eco-research/level-iii-and-iv-ecoregions-continental-united-states (2013).

[37] Hammond TT, Springthorpe D, Walsh RE, Berg-Kirkpatrick T (2016). Using accelerometers to remotely and automatically characterize behavior in small animals. J. Exp. Biol..

[38] Brown DD, Kays R, Wikelski M, Wilson R, Klimley AP (2013). Observing the unwatchable through acceleration logging of animal behavior. Anim. Biotelemetry.

[39] Campbell HA, Goa L, Bidder OR, Hunter J, Franklin CE (2013). Creating a behavioural classification module for acceleration data: Using a captive surrogate for difficult to observe species. J. Exp. Biol..

[40] Shamoun-Baranes J (2012). From sensor data to animal behaviour: An oystercatcher example. PLoS ONE.

[41] Blumstein DT, Daniel JC (2007). Quantifying Behavior the JWatcher Way.

[42] Resheff YS, Rotics S, Harel R, Spiegel O, Nathan R (2014). AcceleRater: A web application for supervised learning of behavioral modes from acceleration measurements. Move. Ecol..

[43] Schafer TLJ, Wikle CK, VonBank JA, Ballard BM, Weegman MD (2020). A Bayesian Markov model with Pólya-Gamma sampling for estimating individual behavioral transition probabilities from accelerometer classifications. J. Agric. Biol. Environ. Stat..

[44] Holsclaw T, Greene AM, Robertson AW, Smyth P (2017). Bayesian nonhomogeneous Markov models via Pólya-Gamma data augmentation with applications to rainfall modeling. Ann. Appl. Stat..

[45] Polson NG, Scott JG, Windle J (2013). Bayesian inference for logistic models using Pólya-Gamma latent variables. J. Am. Stat. Assoc..

[46] Leos-Barajas V (2017). Analysis of animal accelerometer data using hidden Markov models. Methods Ecol. Evol..

[47] Brooks SP, Gelman A (1998). General methods for monitoring convergence of iterative simulations. J. Comput. Graph. Stat..

[48] Geweke, J. *Evaluating the Accuracy of Sampling-Based Approaches to Calculating Posterior Moments. Bayesian Statistics 4*. (Oxford University Press, 1992).

[49] Plummer M, Best N, Cowles K, Vines K (2006). CODA: Convergence diagnosis and output analysis for MCMC. R News.

[50] Bose S, Forrester TD, Casady DS, Wittmer HU (2018). Effect of activity states on habitat selection by black-tailed deer. J. Wildl. Manag..

[51] Cooper, A. B., & Millspaugh, J. J. Accounting for variation in resource availability and animal behavior in resource selection studies. In *Radio Tracking and Animal Populations* (eds. Millspaugh, J. J. & Marzluff, J. M.) 243–273 (Academic Press, 2001).

[52] Zeller KA (2014). Sensitivity of landscape resistance estimates based on point selection functions to scale and behavioral state: Pumas as a case study. Landsc. Ecol..

[53] Abrahms B (2015). Lessons from integrating behaviour and resource selection: Activity-specific responses of African wild dogs to roads. Anim. Conserv..

[54] Séchaud R (2021). Behavior-specific habitat selection patterns of breeding barn owls. Move. Ecol..

[55] Boyce MS (2006). Scale for resource selection functions. Divers. Distrib..

[56] Pohle J, Langrock R, van Beest FM, Schmidt NM (2017). Selecting the number of states in hidden Markov models: pragmatic solutions illustrated using animal movement. J. Agric. Biol. Environ. Stat..

[57] Buderman FE (2021). Caution is warranted when using animal space-use and movement to infer behavioral states. Move. Ecol..

[58] Davis JB (2014). Habitat and resource use by waterfowl in the northern hemisphere in autumn and winter. Wildfowl Spec. Issue.

[59] Dorak BE (2017). Survival and habitat selection of Canada geese during autumn and winter in metropolitan Chicago, USA. Condor.

[60] Calder, W. A., King, J. R. Thermal and caloric relations of birds. In *Avian Biology*, vol. IV (eds. Farner, D. S. & King, J. R.) 259–413 (Academic Press, 1974).

[61] Jónsson JE, Afton AD (2009). Time budgets of Snow Geese *Chen caerulescens* and Ross's Geese *Chen rossii* in mixed flocks: Implications of body size, ambient temperature and family associations. Ibis.

[62] Kölzsch A (2016). Neckband or backpack? Differences in tag design and their effects of GPS/accelerometer tracking results in large waterbirds. Anim. Biotelemetry.

[63] Morrison, M. L., Brennan, L. A., Marcot, B. G., Block, W. A., & McKelvey, K. S. *Applications for Advancing Animal Ecology* (Johns Hopkins University Press, 2021).

