# Supplementary material for: Joint use of location and acceleration data reveals influences on transitions among habitats in wintering birds
Source: Sci Rep. 2023 Feb 6;13:2132. doi: 10.1038/s41598-023-28937-x (PMC9902612; doi:10.1038/s41598-023-28937-x)
Supplement: Supplementary file 1 — Supplementary Information. [file 41598_2023_28937_MOESM1_ESM.docx]

**Electronic Supplementary Material**

**Joint use of location and acceleration data reveals influences on transitions among habitats in wintering birds**

Jay A. VonBank^1^, Toryn L. J. Schafer^2^, Stephanie A. Cunningham^3^, Mitch D. Weegman^4^, Paul T. Link^5^, Kevin J. Kraai^6^, Christopher K. Wikle^2^, Daniel P. Collins^7^, Lei Cao^8,9^, and Bart M. Ballard^1^

^1^Caesar Kleberg Wildlife Research Institute, Texas A&M University – Kingsville, Kingsville, TX, 78363, USA

^2^Department of Statistics, University of Missouri, Columbia, MO, 65211, USA.

^3^School of Natural Resources, University of Missouri, Columbia, MO, 65211, USA.

^4^Department of Biology, University of Saskatchewan, Saskatoon, SK, S7N 5E2, Canada.

^5^Louisiana Department of Wildlife and Fisheries, Baton Rouge, LA, 70808, USA.

^6^Texas Parks and Wildlife Department, Canyon, TX, 79015, USA.

^7^U.S. Fish and Wildlife Service Region 2, Albuquerque, NM, 87102, USA.

^8^State Key Laboratory of Urban and Regional Ecology, Research Center for Eco-Environmental Sciences, Chinese Academy of Sciences, 100085, Beijing, China

^9^University of Chinese Academy of Sciences, 100049, Beijing, China

**D**

**C**

**B**

**A**


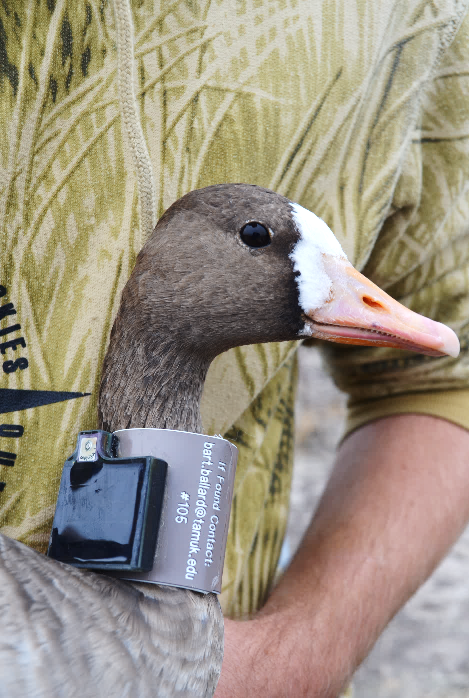

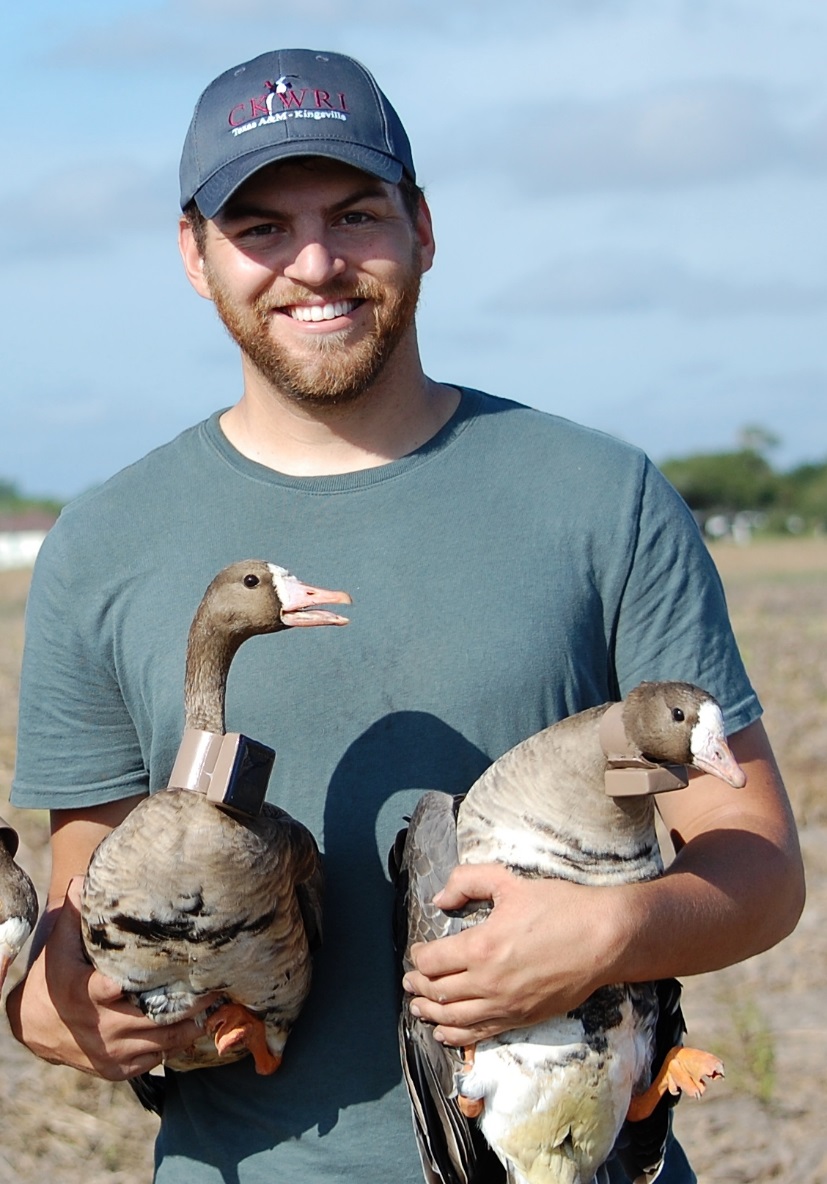

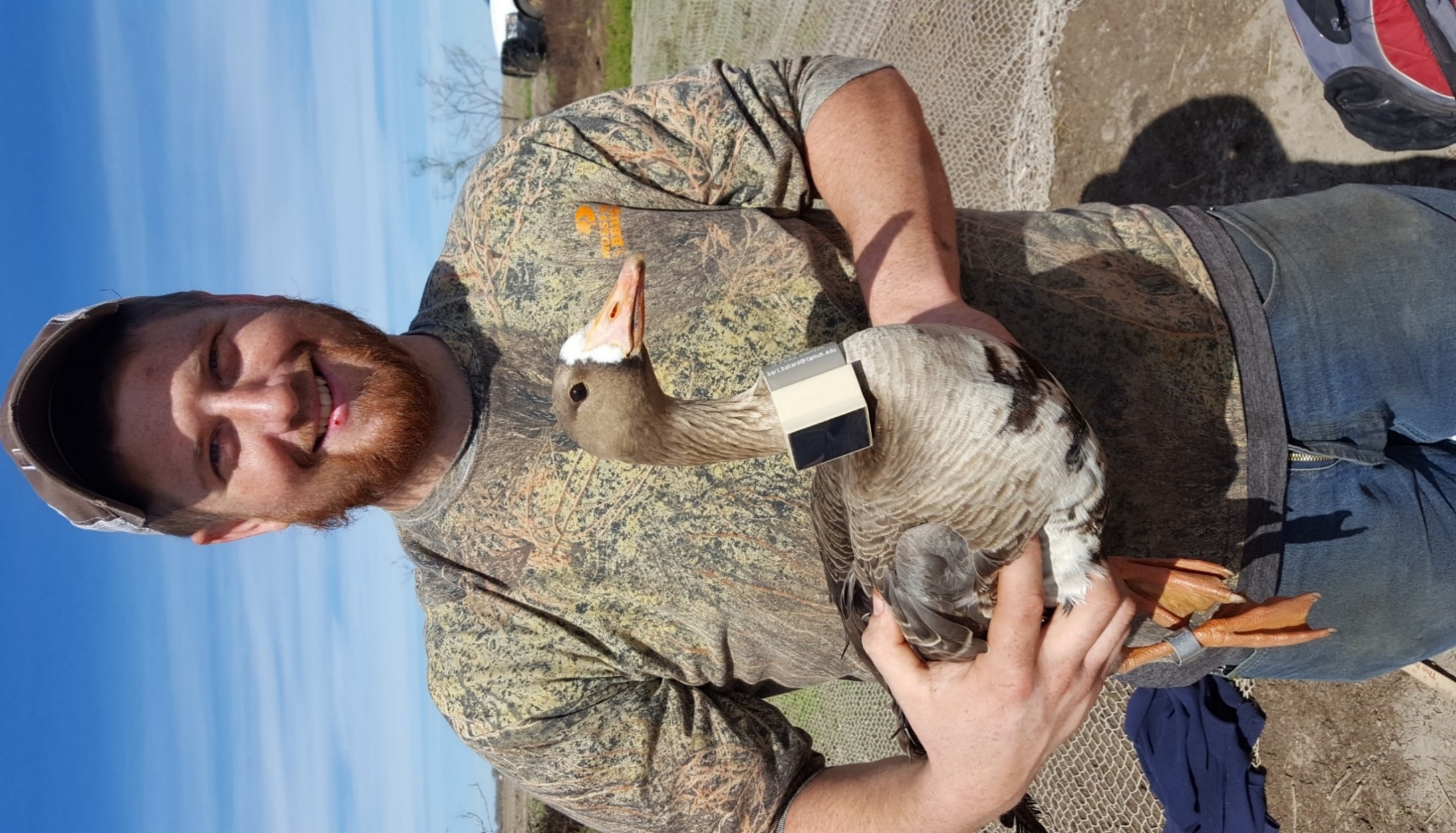

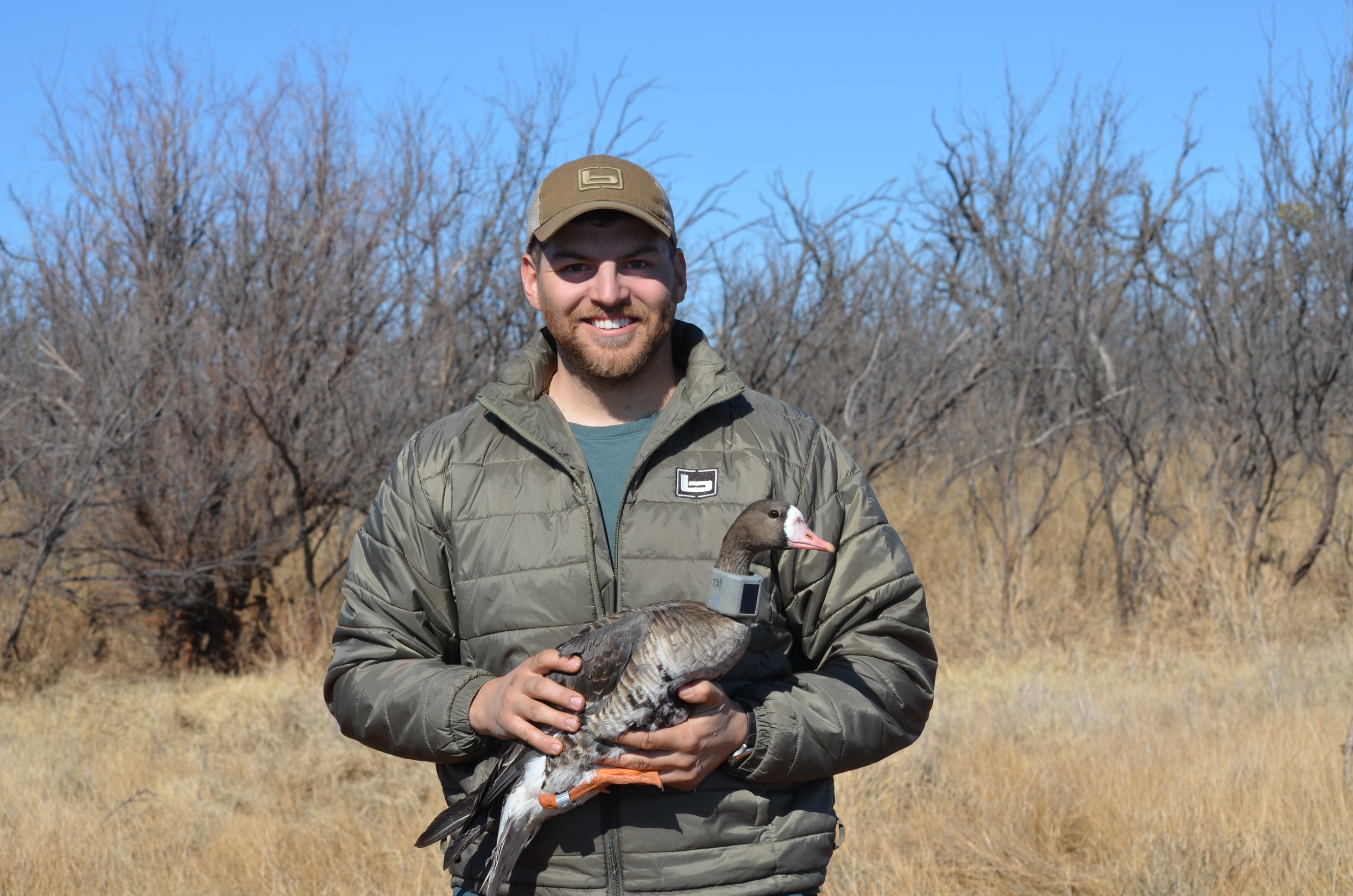


**Figure S1.** Four GPS/ACC/GSM device types deployed on a total of 56 greater white-fronted geese (*Anser albifrons frontalis*) from October through February, 2016-2017 and 2017-2018. Devices models included Cellular Tracking Technologies A) BT 3.0, B) BT 3.5, and C) BT 3.75, and Ornitela device D) OrniTrack-N38.


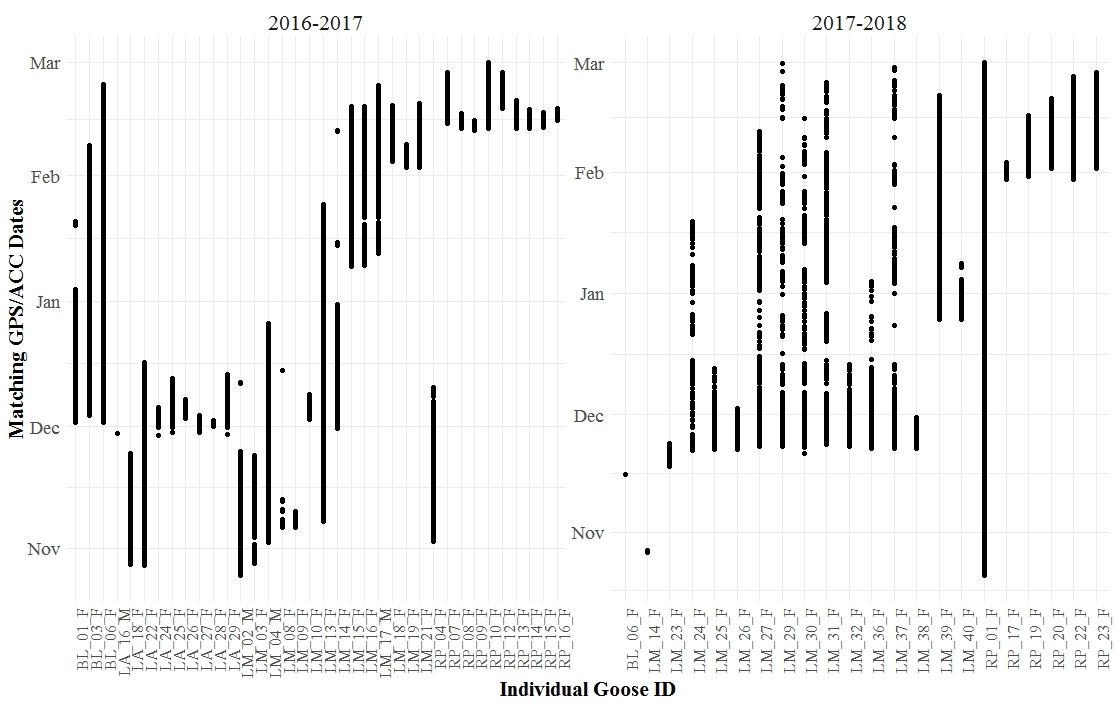


**Figure S2.** Time series of matched ACC-GPS data from individual greater white-fronted geese (*Anser albifrons frontalis*) across winters 2016-2017 and 2017-2018 used in the Bayesian Markov model to estimate habiat transitions influenced by behavior. Gaps in individual time series represent either missing GPS or missing ACC data.

**Table S1.** Original land cover types and their database origin which comprised categorized habitat types used in the analysis of habitat transitions by greater white-fronted geese (*Anser albifrons frontalis*)

|  | Habitat Class Spatial Layer Origin | |
| --- | --- | --- |
| Categorized Habitat Type | CDL^a^ | C-CAP^b^ |
| Corn | Corn, Sweet Corn | — |
| Grass/Winter Wheat | Alfalfa, Grass/Pasture, Other Hay/Non Alfalfa, Sod/Grass Seed, Winter Wheat, Spring Wheat, Dbl Crop WinWht/Corn, Dbl Crop WinWht/Cotton, Dbl Crop WinWht/Sorghum, Dbl Crop WinWht/Soybeans | — |
| Herbaceous Wetland | Herbaceous Wetlands | Estuarine Aquatic Bed, Estuarine Emergent Wetland |
| Other | Cotton, Fallow/Idle Cropland, Developed/Open Space, Developed/Low Intensity, Developed/Med Intensity, Developed/High Intensity, Barley, Canola, Greens, Herbs, Millet, Oats, Other Crops, Peas, Rye, Sugarcane, Sunflower, Triticale | — |
| Other Grain | Peanuts^c^, Sorghum, Soybeans | — |
| Rice | Rice, Aquaculture | — |
| Woody Wetlands | Woody Wetlands | — |
| Open Water/Unconsolidated Shore | Open Water, Barren | Open Estuarine Water, Unconsolidated Shore |
| ^a^ National Agricultural Statistics Service Cropland Data Layer.  ^b^ National Oceanic and Atmospheric Administration, Office for Coastal Management. Coastal Change Analysis Program (C-CAP) Regional Land Cover.  ^c^ Peanut classification was derived from Landsat 8 satellite imagery and unsupervised classification. | | |

**Table S2.** Numer of GPS locations for each of eight habitat types and seven ecoregions used by GPS tracked greater white-fronted geese (*Anser albifrons frontalis*) for each winter 2016-2017, 2017-2018, and for the total study period combined.

| Winter 2016-2017 | Region | | | | | | |  |
| --- | --- | --- | --- | --- | --- | --- | --- | --- |
| Habitat Type | Chenier Plain | Lower  Gulf Coast | MAV | Other | Rolling/ High Plains | STX Brushlands | Texas Mid-coast | Total Habitat Locations |
| Corn | 0 | 291 | 168 | 8 | 0 | 426 | 178 | 1,071 |
| Grass/Winter Wheat | 26 | 234 | 1 | 8 | 204 | 773 | 25 | 1,271 |
| Herbaceous Wetlands | 582 | 292 | 0 | 0 | 1 | 371 | 49 | 1,295 |
| Other | 192 | 457 | 73 | 16 | 35 | 720 | 597 | 2,090 |
| Other Grain | 20 | 3,154 | 195 | 8 | 233 | 1,717 | 109 | 5,436 |
| Rice | 2,386 | 0 | 1,010 | 91 | 0 | 0 | 804 | 4,291 |
| Woody Wetlands | 0 | 679 | 45 | 9 | 1,401 | 7,845 | 0 | 9,979 |
| Open Water/Unconsol. | 28 | 3,450 | 158 | 1 | 279 | 2,135 | 92 | 6,143 |
| Total Region Locations | 3,234 | 8,557 | 1,650 | 141 | 2,153 | 13,987 | 1,854 | 31,576 |
|  |  |  |  |  |  |  |  |  |
| Winter 2017-2018 | Region | | | | | | |  |
| Habitat Type | Chenier Plain | Lower  Gulf Coast | MAV | Other | Rolling/ High Plains | STX  Brushlands | Texas  Mid-coast | Total Habitat Locations |
| Corn | 0 | 231 | 52 | 1,115 | 0 | 1 | 165 | 1,564 |
| Grass/Winter Wheat | 25 | 153 | 7 | 1,957 | 473 | 66 | 219 | 2,900 |
| Herbaceous Wetlands | 8 | 294 | 0 | 374 | 443 | 13 | 312 | 1,444 |
| Other | 124 | 248 | 106 | 94 | 169 | 487 | 353 | 1,581 |
| Other Grain | 14 | 491 | 383 | 570 | 720 | 1,269 | 92 | 3,539 |
| Rice | 133 | 0 | 6 | 247 | 0 | 0 | 499 | 885 |
| Woody Wetlands | 0 | 328 | 21 | 38 | 39 | 8 | 0 | 434 |
| Open Water/Unconsol. | 0 | 3,095 | 31 | 3,450 | 2,362 | 286 | 355 | 9,579 |
| Total Region Locations | 304 | 4,840 | 606 | 7,845 | 4,206 | 2,130 | 1,995 | 21,926 |

**Table S2 Continued.** Numer of GPS locations for each of eight habitat types and seven ecoregions used by GPS tracked greater white-fronted geese (*Anser albifrons frontalis*) for each winter 2016-2017, 2017-2018, and for the total study period combined.

| Total Study | Region | | | | | | |  |
| --- | --- | --- | --- | --- | --- | --- | --- | --- |
| Habitat Type | Chenier Plain | Lower  Gulf Coast | MAV | Other | Rolling/ High Plains | STX  Brushlands | Texas  Mid-coast | Total Habitat Locations |
| Corn | 0 | 522 | 220 | 1,123 | 0 | 427 | 343 | 2,635 |
| Grass/Winter Wheat | 51 | 387 | 8 | 1,965 | 677 | 839 | 244 | 4,171 |
| Herbaceous Wetlands | 590 | 586 | 0 | 374 | 444 | 384 | 361 | 2,739 |
| Other | 316 | 705 | 179 | 110 | 204 | 1,207 | 950 | 3,671 |
| Other Grain | 34 | 3,645 | 578 | 578 | 953 | 2,986 | 201 | 8,975 |
| Rice | 2,519 | 0 | 1,016 | 338 | 0 | 0 | 1,303 | 5,176 |
| Woody Wetlands | 0 | 1,007 | 66 | 47 | 1,440 | 7,853 | 0 | 10,413 |
| Open Water/Unconsol. | 28 | 6,545 | 189 | 3,451 | 2,641 | 2,421 | 447 | 15,722 |
| Total Region Locations | 3,538 | 13,397 | 2,256 | 7,986 | 6,359 | 16,117 | 3,849 | 53,502 |


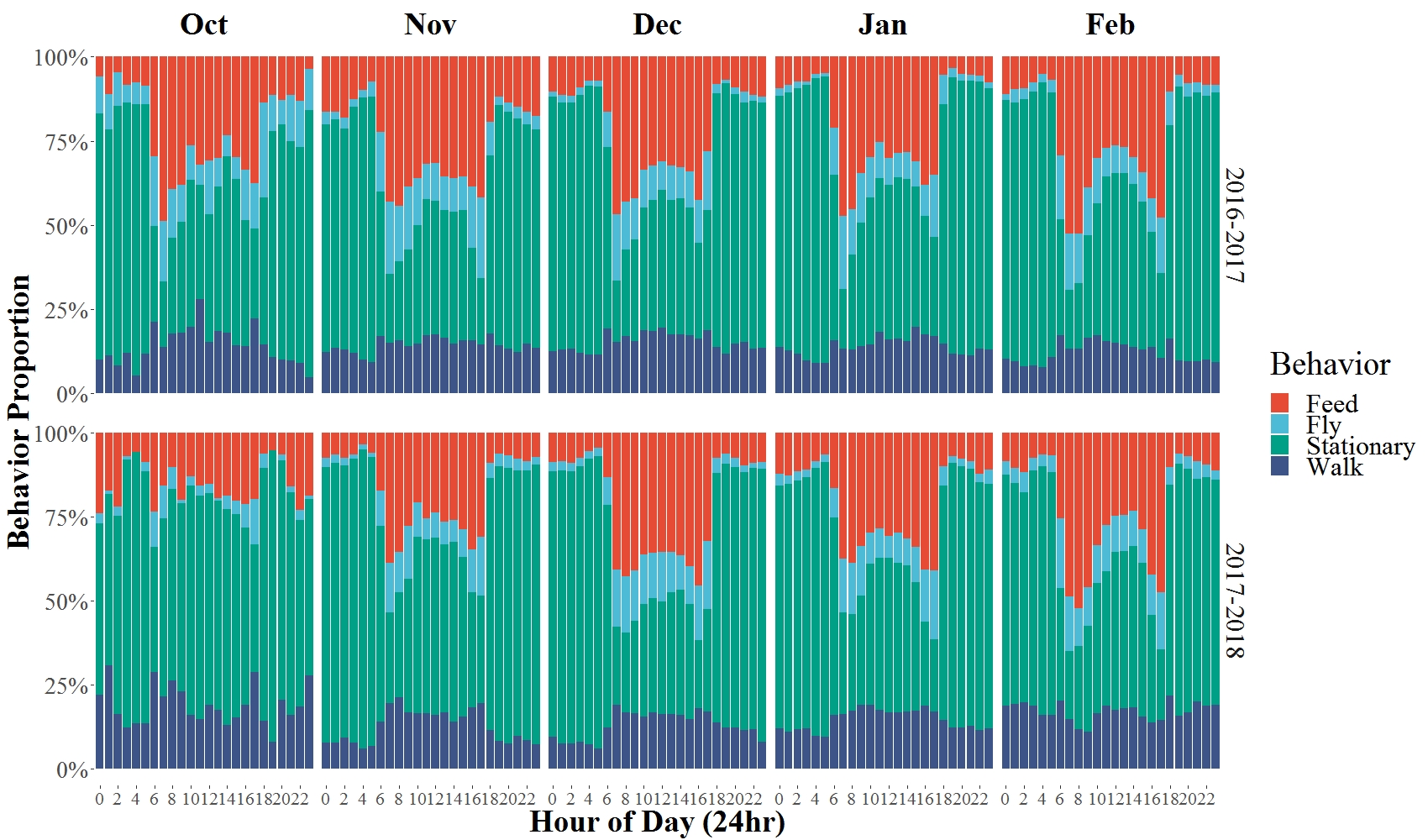


**Figure S3.** Hourly time-activity budgets from 300,348 behavior bursts of 56 greater white-fronted geese across 7 ecoregions from October through February, 2016-2017 and 2017-2018. Behaviors were classified by ground-truthed acceleration signatures from captive and wild greater white-fronted geese, and acceleration data from wild individuals was classified by random forest machine learning algorithm.


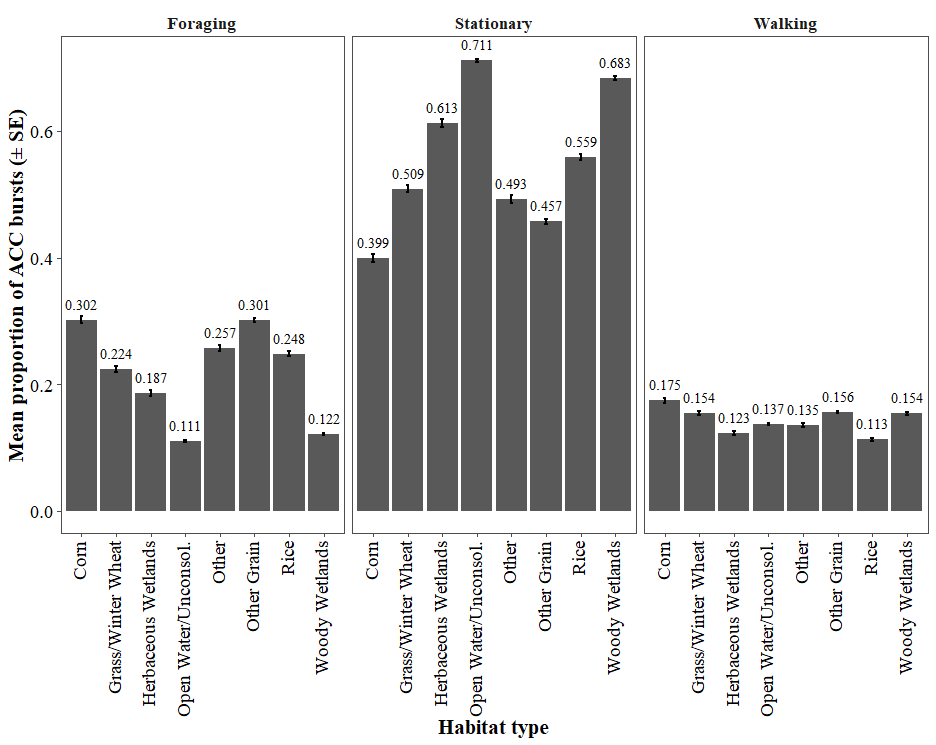


**Figure S4.** Mean proportion (± SE) of accelerometer (ACC) bursts classified as foraging, stationary, or walking within each of eight habitat types used by 56 greater white-fronted geese across 7 ecoregions from October through February, 2016-2017 and 2017-2018.
